# Efficacy and Safety of 5% Minoxidil Alone, Minoxidil Plus Oral Spironolactone, and Minoxidil Plus Microneedling on Female Pattern Hair Loss: A Prospective, Single-Center, Parallel-Group, Evaluator Blinded, Randomized Trial

**DOI:** 10.3389/fmed.2022.905140

**Published:** 2022-07-11

**Authors:** Xuelei Liang, Yuan Chang, Haixuan Wu, Yi Liu, Jian Zhao, Leyi Wang, Fenglin Zhuo

**Affiliations:** Department of Dermatology, Beijing Friendship Hospital, Capital Medical University, Beijing, China

**Keywords:** female pattern hair loss (FPHL), microneedling, spironolactone, minoxidil, efficacy and safety

## Abstract

**Background:**

The efficacy of topical minoxidil (MX) alone on female pattern hair loss (FPHL) is limited. Combination therapy based on topical MX is currently expected to provide better outcomes.

**Objectives:**

This study aimed to assess whether the combined therapies including MX plus oral spironolactone (SPT) and MX plus microneedling (MN) have advantages in efficacy and safety over topical MX alone on mild-to-moderate FPHL with normal hormone levels in the blood and regular menstrual cycle.

**Methods:**

A prospective, single-center, parallel-group, evaluator blinded, randomized trial including 120 non-menopause women with proven FPHL (Sinclair class II-III) was performed in China. Patients were randomly assigned to three groups, namely, the MX group (5% topical MX alone, once daily), the MX + SPT group (MX plus SPT 80–100 mg daily), and the MX+MN group (MX plus MN every 2 weeks, 12 sessions). The change from the baseline to week 24 was assessed in hair growth (hair density and diameter under dermoscope), scalp tissue structure (epidermal thickness, dermis thickness, and average hair follicle diameter under ultrasound biomicroscopy), physician's global assessment (using a 7-point global-assessment scale and Sinclair's stage change), patient evaluation (Women's Androgenetic Alopecia Quality of Life Questionnaire and Sinclair's hair-shedding score) and side effects.

**Results:**

In total, 115 participants completed the trial. At week 24, the hair density increased most in MX + MN group and increased least in MX group (*p* < 0.001 for MX + MN group vs. MX + SPT group; *p* = 0.009 for MX + SPT group vs. MX group). The hair shaft diameter significantly increased in all groups (*p* < 0.001, respectively), but there were no significant differences among the three groups (*p* = 0.905). The epidermal thickness and average hair follicle diameter only increased in MX + MN group. Dermis thickness increased in all groups, but there were no significant differences among the three groups. Both physician's and patient assessments showed improvement in all three groups. Scalp pruritus was the most common side effect. The MX + SPT group had the most reported adverse effects.

**Limitations:**

The main limitations of this study are the relatively small sample size, the exclusion of severe FPHL patients, and the potential bias from unblinded treatments among the 3 groups.

**Conclusion:**

Topical MX combined with MN is a better choice than either MX plus oral SPT or MX alone for the treatment of mild-to-moderate FPHL patients.

## Introduction

Female pattern hair loss (FPHL) used to be called androgenic alopecia (AGA) including both men and women. However, due to the differences in clinical features, FPHL is more widely used to describe AGA in women (1). FPHL is the most common form of alopecia in women and its prevalence increases with age (2). It was reported that 19.0% of Caucasian women (3), 5.6% of Korean women (4), and 6.0% of Chinese women (2) suffer from FPHL. Women affected with AGA will experience more psychological distress and impaired social functioning than men affected with AGA. So, FPHL patients deserve much attention and better therapies (5).

Although the role of androgens in the pathogenesis of male hair loss has been clearly established, the etiology of FPHL is still unclear, which is thought to be polygenic and multifactorial with the additional influence of environmental factors. The effective and safe treatments of FPHL remain limited. So far, topical minoxidil (MX) is the only Food and Drug Administration (FDA)-approved drug for FPHL (6). However, only approximately 40% of patients showed a significant improvement after 3 to 6 months of MX application (7). Thus, new treatments are urgently needed. In recent decades, more and more emerging treatments appeared but are mostly used in men. Several studies have reported that oral spironolactone (SPT) and microneedling (MN) are effective and well-tolerated options for FPHL as adjunct therapy (8, 9).

Off-labeled SPT is an antiandrogen agent. A recent retrospective study showed that greater improvement by oral SPT was observed in severe FPHL patients than in mild or modest ones (8). Nevertheless, adverse events limited its wide application. MN can create a minimally invasive channel to puncture the stratum corneum which induces collagen formation, neovascularization, and growth factor production in treated areas. MN combined with topical MX solution for male AGA is recommended in recent years (10). However, there are no related randomized controlled trials on FPHL.

In this study, we conducted a randomized trial comparing the effectiveness and safety of the three regimens consisting of topical MX alone, MX plus oral SPT, and MX plus MN on FPHL patients with normal hormone levels in the blood and regular menstrual cycle. The aim of this study was to assess whether the combined therapies could have advantages in efficacy and safety over topical MX alone. Also, the best regimen for mild or moderate FPHL with a regular menstrual cycle would be first suggested.

## Materials and Methods

### Study Design and Participants

A prospective, single-center, parallel-group, evaluator blinded, randomized trial was performed in the Department of Dermatology at Beijing Friendship Hospital, Capital Medical University in China. The Ethics Committee of our hospital approved the study with the number 2020-P2-248-01. The clinical trial registry number of this study is ChiCTR2100044015 (https://www.chictr.org.cn/showprojen.aspx?proj=29,280). All patients were informed of the risks, benefits, and possible complications of the process before enrollment in the study, and informed consent was obtained from each patient.

We recruited consecutive FPHL patients in our alopecia clinics from October 2020 to August 2021 if they fulfilled the following criteria: (1) aged 18–45 years; (2) female; (3) with normal hormone levels in the blood and regular menstrual cycle (estradiol, progesterone, prolactin, follicle-stimulating hormone, luteinizing hormone, and testosterone were tested during 2–5 days of the menstrual period. Patients with the hormonal level in the reference range of follicular phase could be enrolled); (4) FPHL diagnosis is proved by Sinclair class for II and III. FPHL was diagnosed based on the previous history of hair loss and the clinical and dermatoscopic appearance of the scalp.

Exclusion criteria included the following: (1) presence of other forms of alopecia; (2) presence of relative contraindications of SPT, such as insufficiency of heart, liver, and kidney, low blood pressure, or elevated blood potassium; (3) presence of relative contraindications of MN, such as a history of bleeding disorders or using anticoagulants medications, active infection at the targeted area, and keloidal tendency; (4) pregnancy or lactation; and (5) use of topical or systemic medications that influence hair growth within the past 6 months. We calculated the sample size required for this trial by the PASS 15.0 software (NCSS, LLC, Kaysville, Utah, United States). Referring to a previous study for male androgenetic alopecia with 5% MX solution two times a day (11), the hair density change from week 24 to baseline was 18.8 ± 9.6 /cm^2^ in the MX group and 38.3 ± 11.1/cm^2^ in MX + MN group, respectively. In addition, we made a hypothesis that the hair density change from baseline to week 24 in MX + SPT group was the average of the MX group and MX + MN group because these data in the real world were not available. We set that α = 0.0167, β = 0.10. The sample size needed for the comparison between the MX group and the MX + MN group is 10 for each group. The sample size needed for the comparison between the MX group and the MX + SPT group is 30 for each group. The sample size needed for the comparison between MX + SPT group and the MX + MN group is 35 for each group. Above all, at least 35 samples for each group were required for statistical analyses. Considering the perhaps 10% loss rate of follow-up, the sample size for each group is 39 (35/0.9).

### Randomization

The participants were randomly assigned to three regimens by using a computer-generated randomization table. Since the therapy differed among the three groups, the study was not masked.

### Procedure

All patients were randomly assigned to the following three groups: (1) MX group: The patients received 1 ml of topical 5% MX tincture (Man Di, ZheJiang WanSheng Pharmaceutical Co., Ltd. China) once daily for 24 weeks; (2) MX + SPT group: The patients received oral SPT of 80–100 mg/day and 1 ml of topical 5% MX once daily for 24 weeks; (3) MX + MN group: The patients received MN treatments with the delivery of 5% MX every 2 weeks and 1 ml of topical 5% MX once daily for 24 weeks. All participants used the same household device with the scale (Scalp Solutions Applicator, Bella Medical Technology, China) to ensure the exact volume applied. Patients in MX + MN group received microneedling treatments according to the following protocol: (1) Patients washed their hair before treatment; (2) alcohol cotton balls were used to clean the treatment sites three times; (3) 1 ml of 5% MX was topically applied; (4) an electrodynamic microneedling was inserted at a depth of 0.7–1.0 mm; (5) the treatment endpoints were punctate hemorrhage and redness at the treatment site; (6) patients were told to not wash their hair within 8 h and not to use MX within 24 h. During the entire study period, no other treatment expected to be effective for FPHL was allowed. The enrolled patients were required to maintain the same hair length and color before any treatment and follow-up. All patients were allowed to drop out of the trial if a severe or life-threatening side effect occurred, or if the treatment did not respond as they expected.

Study visits were scheduled for week 12 and week 24. At baseline, patients' information, including age, body mass index (BMI), age of disease onset, disease duration, and family history, were collected. Moreover, dermoscope evaluation, ultrasound biomicroscopy (UBM), physician's global assessment, and patient assessment were also collected at baseline and each visit.

The most severe zone of alopecia (generally is the mid-point of the connection of the left and right external auditory ear canals) from each patient was marked with a red dot tattoo at baseline to ensure consistency. The tattoo was at the center of the target circle zone, and the diameter of this circle was 1.5 cm. Hair was clipped to a length of 1.6 mm in this zone at baseline and follow-ups. The Dermoscope system (Dermoscopy Image Diagnostic Workstation, Dermat Speedy Recovery T&D Co., Ltd. Beijing, China) was used to take pictures of the target zone under 20–folds magnification. Three board-certified dermatologists blinded to the treatment group evaluated the total hair density and hair shaft diameter according to the dermoscopy images. Hair density was calculated as the number of hairs per cm^2^. The area of the photograph was automatically acquired in the dermoscope system. All hairs in the photograph of the target zone were marked manually and then counted by the cell count function in the ImageJ software. For each hair shaft in the photograph, we clicked and chose the diameter of it using the measuring tool of the dermoscope system, and then, the length was automatically calculated. The average diameter of all hairs in every photograph was recorded. UBM in 50 MHz (MD-310 Skin Ultrasound Biomicroscope, Meda Co., Ltd. Tianjin, China) was used to assess the scalp tissue structure. The examination was done using a specially designed cup (26 mm diameter) that forms a water bath environment of 3 cm height. The epidermal thickness, dermis thickness, and average follicle diameter were measured using the segment tool in the UBM screen. Five hair follicles at the same level were chosen to calculate the average hair follicle diameter.

At baseline and at each visit, we took global photographs by a digital camera (M100, Canon, Japan). The posture and position of patients, the angle and position of the camera, and lighting conditions were well–controlled. Three board-certified dermatologists who were blinded to the treatment group completed the photograph evaluation based on 7-point rating scale (−3 = marked worsening, −2 = moderate worsening, −1 = mild worsening, 0 = no change, 1 = mild improvement, 2 = moderate improvement, 3 = marked improvement). Sinclair's stage change was also assessed. Patient assessments consist of the Women's Androgenetic Alopecia Quality of Life Questionnaire (WAA-QoL) (12) and the Sinclair hair shedding score (6-point scale) (13).

Safety assessment included the recording of all adverse events and laboratory testing of serum potassium levels.

### Statistical Analysis

Continuous variables were recorded as means ± SDs or median (interquartile range, IQR) depending on whether they were a normal distribution. Ordinal variables were recorded as median (IQR). Categorical variables were recorded as a frequency and percentage. The comparison of continuous variables in normal distribution between baseline and follow-ups or among three groups was analyzed with paired *t*-test and one-way ANOVA, respectively. The comparison of continuous variables in abnormal distribution and ordinal variables between baseline and follow-ups or among three groups was analyzed with the Wilcoxon rank-sum test and Kruskal-Wallis test, respectively. Quantitative data were analyzed with the chi-squared test. *P* < 0.05 was considered to be statistically significant. All *P*-values were 2-sided. Particularly, pairwise comparisons among three groups were *post hoc* tests, and thus, *P* < 0.0167 was considered to be statistically significant according to Bonferroni correction. The SPSS 24.0 software (SPSS Inc., Chicago, IL, United States) was used for statistical analysis.

## Results

### Baseline Characteristics

**Figure 1:**
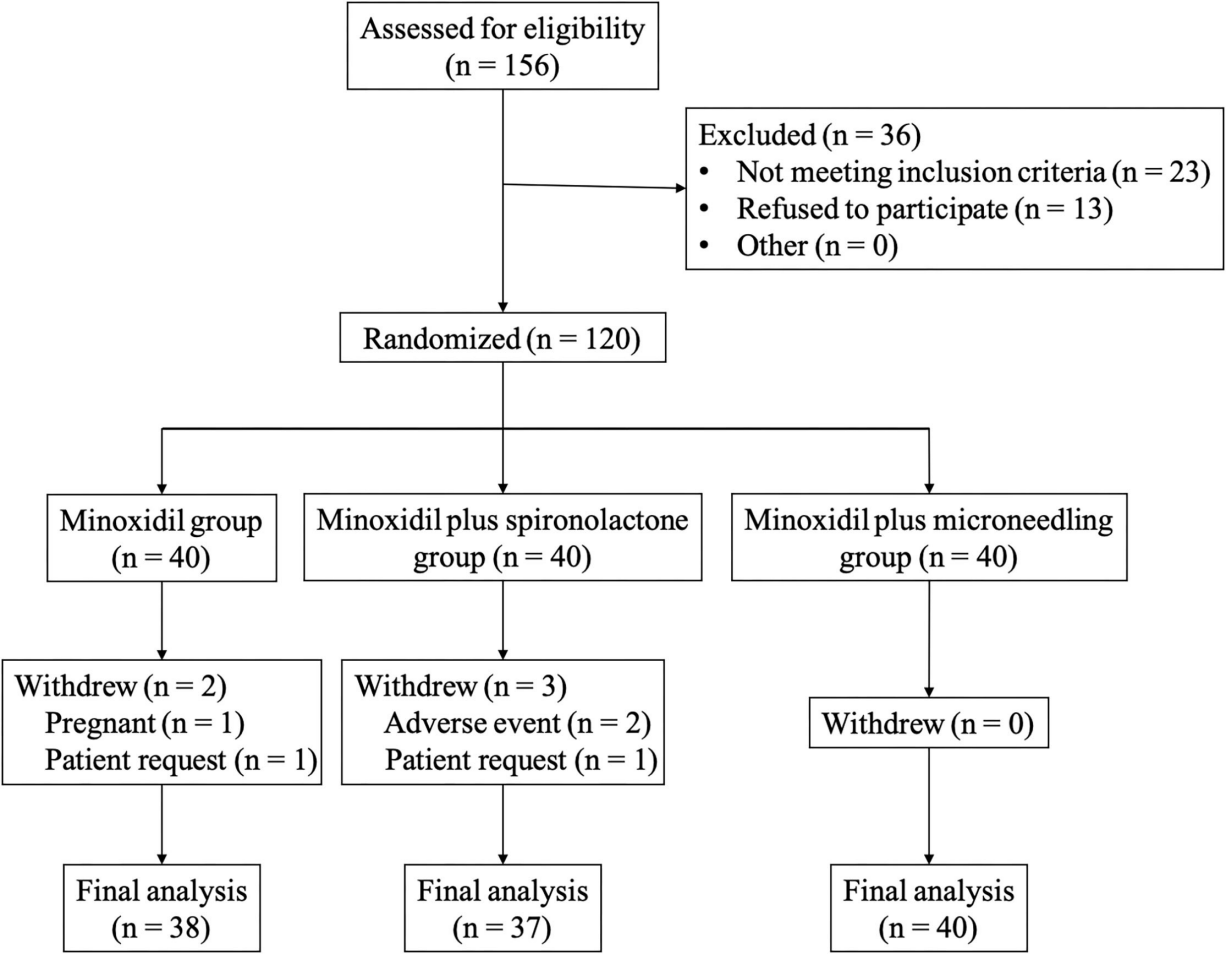
The flowchart of the study.

The flowchart of the study is shown in Figure 1. A total of 120 patients were enrolled in the study with 40 patients in each group. Five patients dropped out of the study. There were no significant differences in demographic and clinical characteristics among the three groups at baseline. Details are shown in Table 1.

**Table 1:** Demographics and baseline characteristics.

**Variable**	**MX**	**MX + SPT**	**MX + MN**	***P*-value**
Age (years)				0.861
mean ± SD	31.08 ± 6.87	31.62 ± 6.29	30.83 ± 6.28	
BMI (kg/m^2^)				0.552
Median (IQR)	21.65(20.25–23.50)	20.90 (19.50–23.85)	21.20 (19.75–22.90)	
Age of onset (years)				0.947
Mean ± SD	25.55 ± 5.44	25.11 ± 6.82	25.43 ± 5.66	
Duration (years)				0.405
Median (IQR)	4.50 (3.00–7.00)	6.00 (3.00–10.00)	5.00 (2.25–8.00)	
Family history				0.337
positive [*n* (%)]	10 (26.32)	15 (40.54)	11 (27.50)	

*MX, minoxidil; SPT, spironolactone, MN: microneedling; SD, standard deviation; BMI, body mass index; IQR, interquartile range; n, the number of patients*.

### Dermoscopic Assessment

Table 2 shows the changes in dermoscopic assessment. There were no significant differences in hair density and hair shaft diameter among the three groups at baseline (*P* = 0.824 for hair density; *P* = 0.597 for hair shaft diameter).

**Table 2:** Dermoscopic assessment during the study period among the three groups.

**Time point**	**Mean ±SD**			**Pairwise comparison *P*-value**			**^♯^*P*-value**
	**MX**	**MX + SPT**	**MX + MN**	**MX** **vs.** **MX +SPT**	**MX** **vs.** **MX + MN**	**MX + SPT** **Vs.** **MX + MN**	
**Hair density (/cm^2^)**							
Baseline	101.37 ± 17.32	99.14 ± 15.85	99.68 ± 15.58	–	–	–	0.824
Week 12	102.47 ± 19.00	111.89 ± 21.10	102.20 ± 16.72	–	–	–	–
Week 24	111.32 ± 19.57	115.89 ± 20.39	130.00 ± 20.58	–	–	–	–
^|^Change	1.11 ± 10.34	12.76 ± 14.34	2.53 ± 13.08	**<0.001**	0.598	**0.002**	**<0.001**
^§^Change	9.95 ± 10.16	16.76 ± 11.75	30.33 ± 15.18	**0.009**	**<0.001**	**<0.001**	**<0.001**
^|^*P*-value	0.514	**<0.001**	0.230	–	–	–	–
^§^*P*-value	**<0.001**	**<0.001**	**<0.001**	–	–	–	–
**Hair shaft diameter (mm)**							
Baseline	44.07 ± 4.79	43.10 ± 4.29	42.85 ± 7.07	–	–	–	0.597
Week 12	51.38 ± 9.82	53.24 ± 6.58	51.03 ± 9.05	–	–	–	–
Week 24	58.43 ± 10.04	58.09 ± 9.32	58.24 ± 11.44	–	–	–	–
^|^Change	7.30 ± 8.89	10.13 ± 6.12	8.18 ± 7.13	0.114	0.632	0.051	0.249
^§^Change	14.36 ± 10.61	14.99 ± 9.25	15.39 ± 10.73	0.785	0.672	0.863	0.905
^|^*P*-value	**<0.001**	**<0.001**	**<0.001**	–	–	–	–
^§^*P*-value	**<0.001**	**<0.001**	**<0.001**	–	–	–	–

*MX, minoxidil; SPT, spironolactone; MN, microneedling; SD, standard deviation.*

| *, week 12 compared with baseline;*

§ *, week 24 compared with baseline;*

♯ *, three groups to compare. Bold value indicates statistical significance*.

The hair density significantly increased only in MX + SPT group (*P* < 0.001) at week 12, while it significantly increased in all three groups at week 24 compared with the baseline (9.95 ± 10.16/cm^2^ in the MX group, 16.76 ± 11.75/cm^2^ in MX + SPT group, and 30.33±15.18/cm^2^ in MX + MN group; *P* < 0.001 for each group from the baseline). As shown, MX + MN group increased in hair density more than both the MX group and MX + SPT group at week 24 (*P* < 0.001). Then, MX + SPT group increased in hair density more than MX alone group (*P* = 0.009).

**Figure 2:**
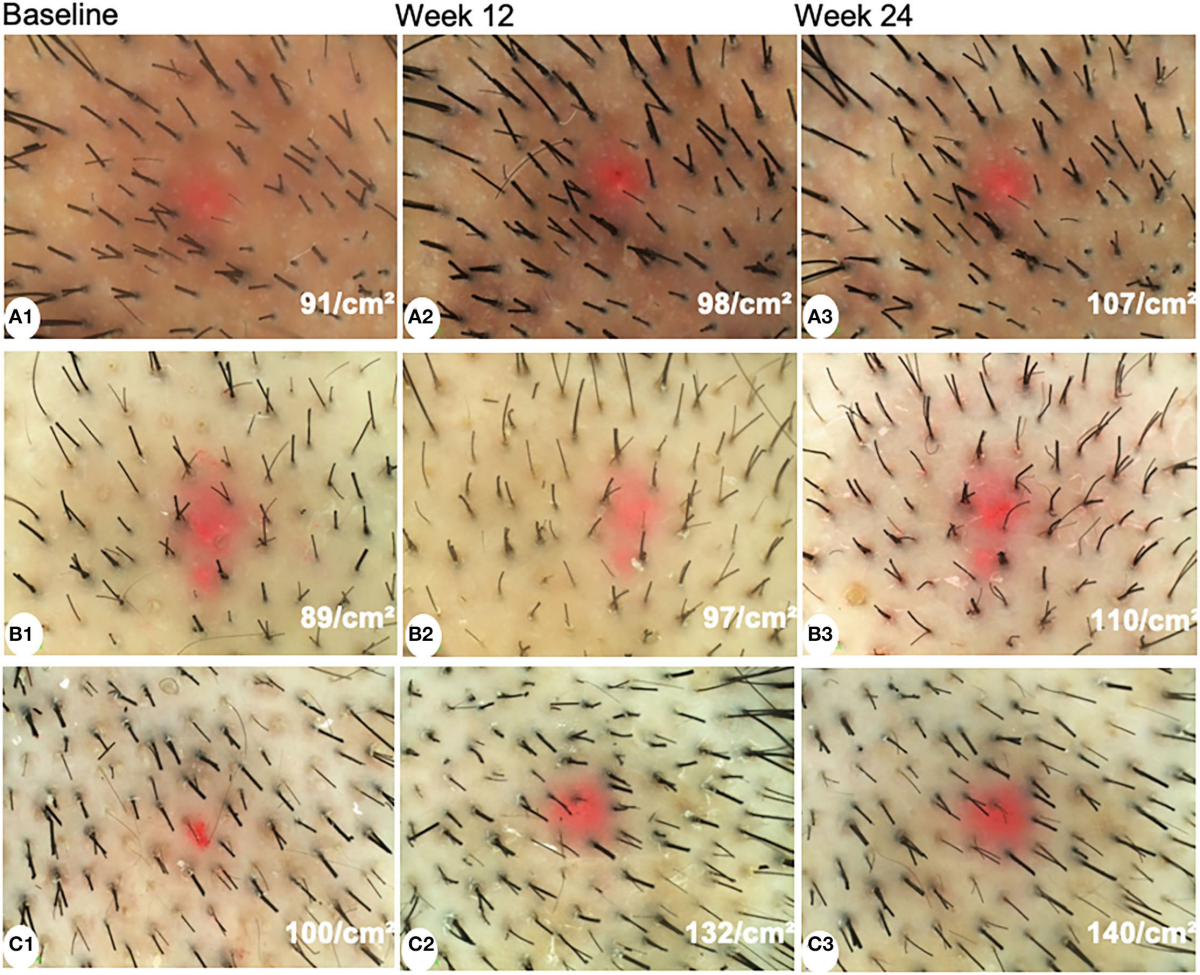
Dermoscopic images of patients treated with minoxidil **(A)**, minoxidil plus spironolactone **(B)**, and minoxidil plus microneedling **(C)** at baseline, week 12, and week 24 (magnification: 20 × ).

The hair shaft diameter significantly increased in all groups both at week 12 and week 24 (*P* < 0.001). However, there were no significant differences among the three groups at week 12 and week 24 (*P* = 0.249 and 0.905, respectively). The representative dermoscopic images before and after treatments are shown in Figure 2.

### UBM Assessment

Table 3 shows the changes in UBM assessment. There were no significant differences in epidermal thickness, dermis thickness, and average follicle diameter among the three groups at baseline (*P* = 0.651, 0.905, and 0.106, respectively).

**Table 3:** UBM assessment during the study period among the three groups.

**Time point**	**Median (IQR)**			**Pairwise comparison *P*-value**			**^♯^*P*-value**
	**MX**	**MX + SPT**	**MX + MN**	**MX** **vs.** **MX + SPT**	**MX** **vs.** **MX +MN**	**MX + SPT** **vs.** **MX + MN**	
**Epidermal thickness (mm)**							
Baseline	0.07 (0.06–0.08)	0.08 (0.06–0.08)	0.08 (0.06–0.09)	–	–	–	0.651
Week 12	0.08 (0.06–0.08)	0.08 (0.07–0.08)	0.09 (0.08–0.10)	–	–	–	–
Week 24	0.08 (0.06–0.08)	0.07 (0.06–0.08)	0.10 (0.08–0.10)	–	–	–	–
^|^Change	0.00 (0.00–0.00)	0.00 (0.00–0.01)	0.01 (0.00–0.02)	0.828	**<0.001**	**<0.001**	**<0.001**
^§^Change	0.00 (0.00–0.01)	0.00 (0.00–0.01)	0.02 (0.01–0.02)	0.904	**<0.001**	**<0.001**	**<0.001**
^|^*P*-value	0.537	0.343	**<0.001**	–	–	–	–
^§^*P*-value	0.088	0.562	**<0.001**	–	–	–	–
**Dermis thickness (mm)**							
Baseline	1.33 (1.17–1.56)	1.39 (1.16–1.57)	1.39 (1.20–1.51)	–	–	–	0.905
Week 12	1.61 (1.48–1.79)	1.76 (1.45–1.95)	1.66 (1.51–1.83)	–	–	–	–
Week 24	1.63 (1.49-1.91)	1.70 (1.49–2.04)	1.82 (1.67–1.97)	–	–	–	–
^|^Change	0.20 (0.06–0.45)	0.19 (0.08–0.75)	0.25 (0.10–0.52)	0.396	0.708	0.911	0.581
^§^Change	0.32 (0.09–0.59)	0.34 (0.16–0.56)	0.46 (0.19–0.69)	0.799	0.587	0.380	0.524
^|^*P*-value	**<0.001**	**<0.001**	**<0.001**	–	–	–	–
^§^*P*-value	**<0.001**	**<0.001**	**<0.001**	–	–	–	–
**Average follicle diameter (mm)**							
Baseline	0.09 (0.08–0.10)	0.08 (0.08–0.09)	0.09 (0.08–0.10)	–	–	–	0.106
Week 12	0.09 (0.09–0.10)	0.09 (0.08–0.10)	0.10 (0.09–0.11)	–	–	–	–
Week 24	0.09 (0.09–0.10)	0.09 (0.08–0.10)	0.10 (0.09–0.11)	–	–	–	–
^|^Change	0.00 (−0.01–0.01)	0.00 (−0.01–0.01)	0.01 (0.00–0.01)	0.427	**0.002**	0.213	**0.021**
^§^Change	0.00 (−0.01–0.01)	0.01 (0.00–0.02)	0.01 (0.01–0.02)	0.307	**<0.001**	**0.013**	**0.001**
^|^*P*-value	0.238	0.163	**<0.001**	–	–	–	–
^§^*P*-value	0.436	0.056	**<0.001**	–	–	–	–

*UBM, Ultrasound biomicroscopy; MX, minoxidil; SPT, spironolactone; MN, microneedling; IQR, interquartile range.*

| *, week 12 compared with baseline;*

§ *, week 24 compared with baseline;*

♯ *, three groups to compare. Bold value indicates statistical significance*.

The epidermal thickness significantly increased only in MX + MN group both at week 12 and week 24 (*P* < 0.001). However, there were no significant changes in the epidermal thickness in the MX group and MX + SPT group during the follow-up (*P* =0.537 and 0.088 for the MX group at week 12 and week 24; *P* = 0.343 and 0.562 for MX + SPT group at week 12 and week 24).

The dermis thickness of the three groups all significantly increased both at week 12 and week 24 (*P* < 0.001). There were no significant differences among the three groups at week 12 and week 24 (*P* = 0.581 and 0.524, respectively).

**Figure 3:**
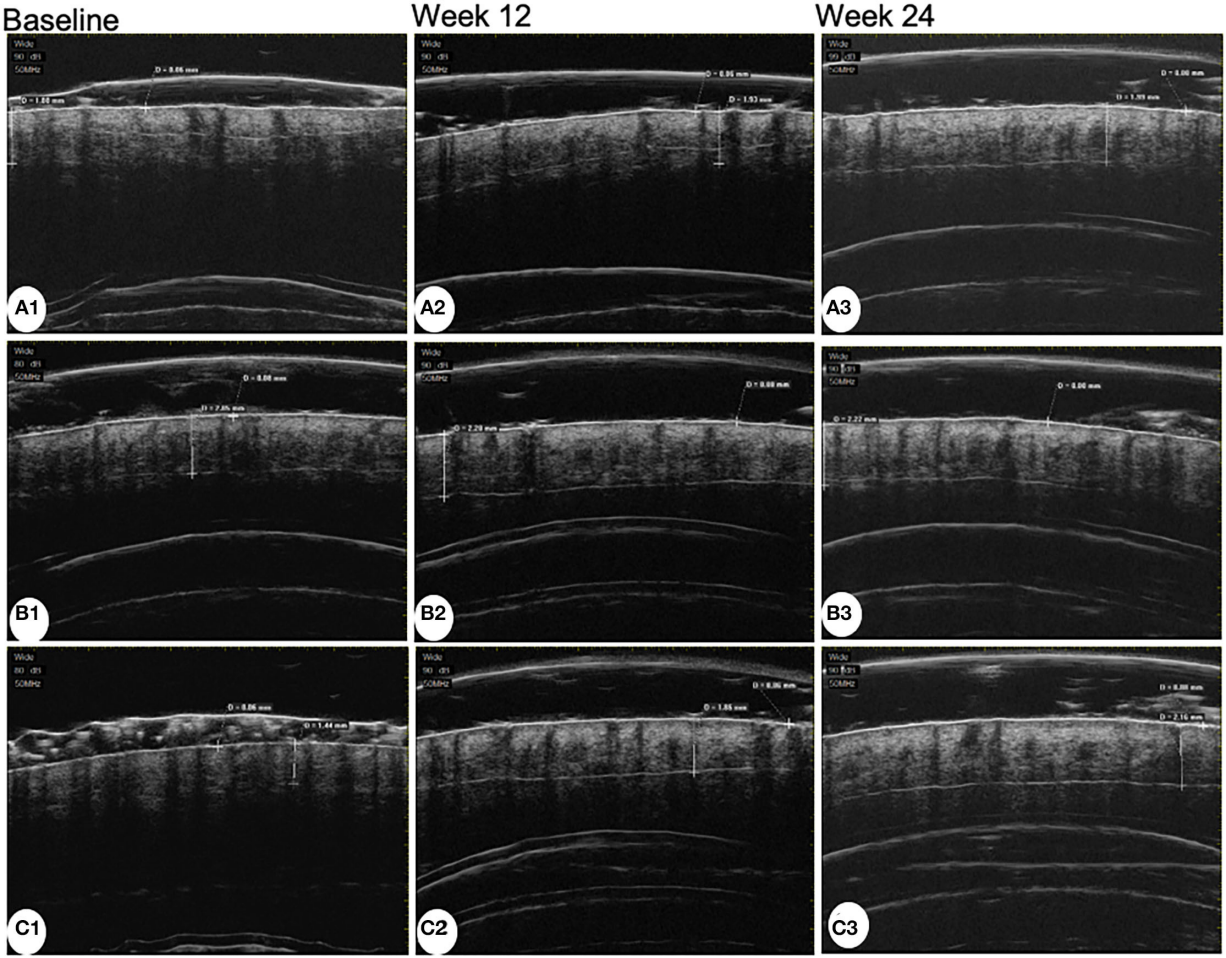
Ultrasound biomicroscopy images of patients treated with minoxidil **(A)**, minoxidil plus spironolactone **(B)**, and minoxidil plus microneedling **(C)** at baseline, week 12, and week 24.

The average follicle diameter of the MX + MN group significantly increased both at week 12 and week 24 (*P* < 0.001). The average follicle diameter had no significant changes in the MX group and MX + SPT group during the follow-up (*P* =0.238 and 0.436 for the MX group at week 12 and week 24; *P* = 0.163 and 0.056 for MX + SPT group at week 12 and week 24). The representative UBM images before and after treatments are shown in Figure 3.

### Investigator Assessment of Hair Growth

**Figure 4:**
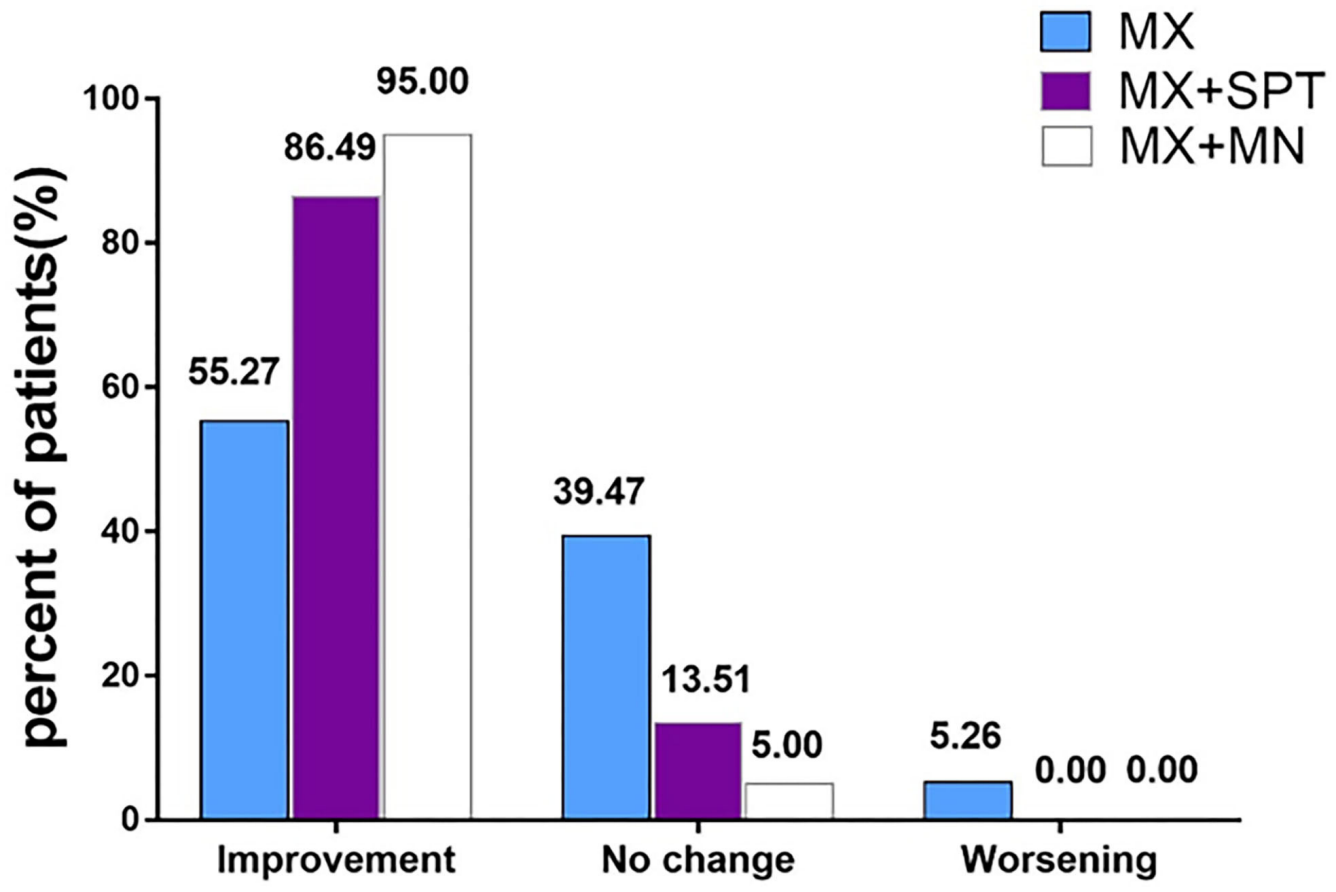
Physician's global photographic assessment based on a standardized 7-point scale at week 24. MX, minoxidil; SPT, spironolactone; MN, microneedling.

**Figure 5:**
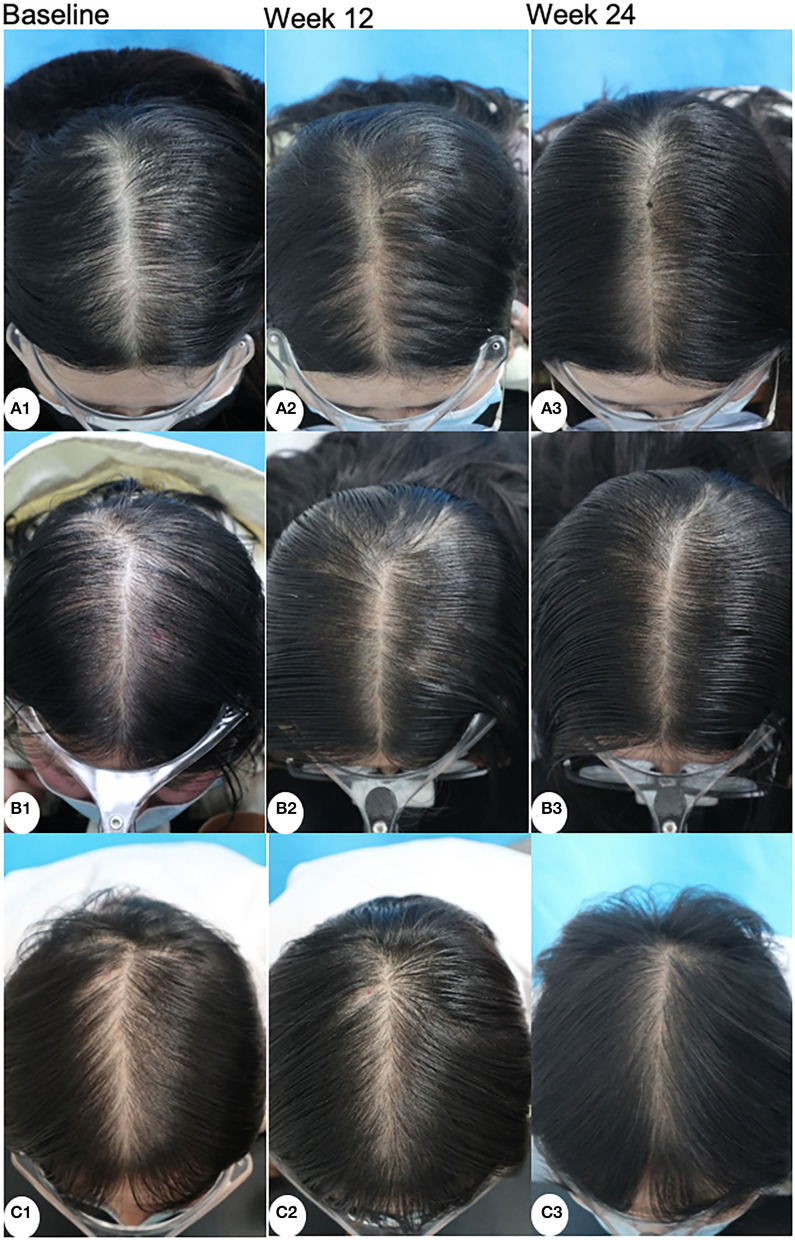
Clinical photographs of patients treated with minoxidil **(A)**, minoxidil plus spironolactone **(B)**, and minoxidil plus microneedling **(C)** at baseline, week 12, and week 24.

Assessed by investigators according to global photographs, 55.27% of patients in the MX group, 86.49% of patients in the MX + SPT group, and 95.00% of patients in the MX + MN group showed improvement at week 24 (Figure 4). Among the three groups, only 5.26% of patients in the MX group got worse. Clinical photographs are shown in Figure 5.

**Figure 6:**
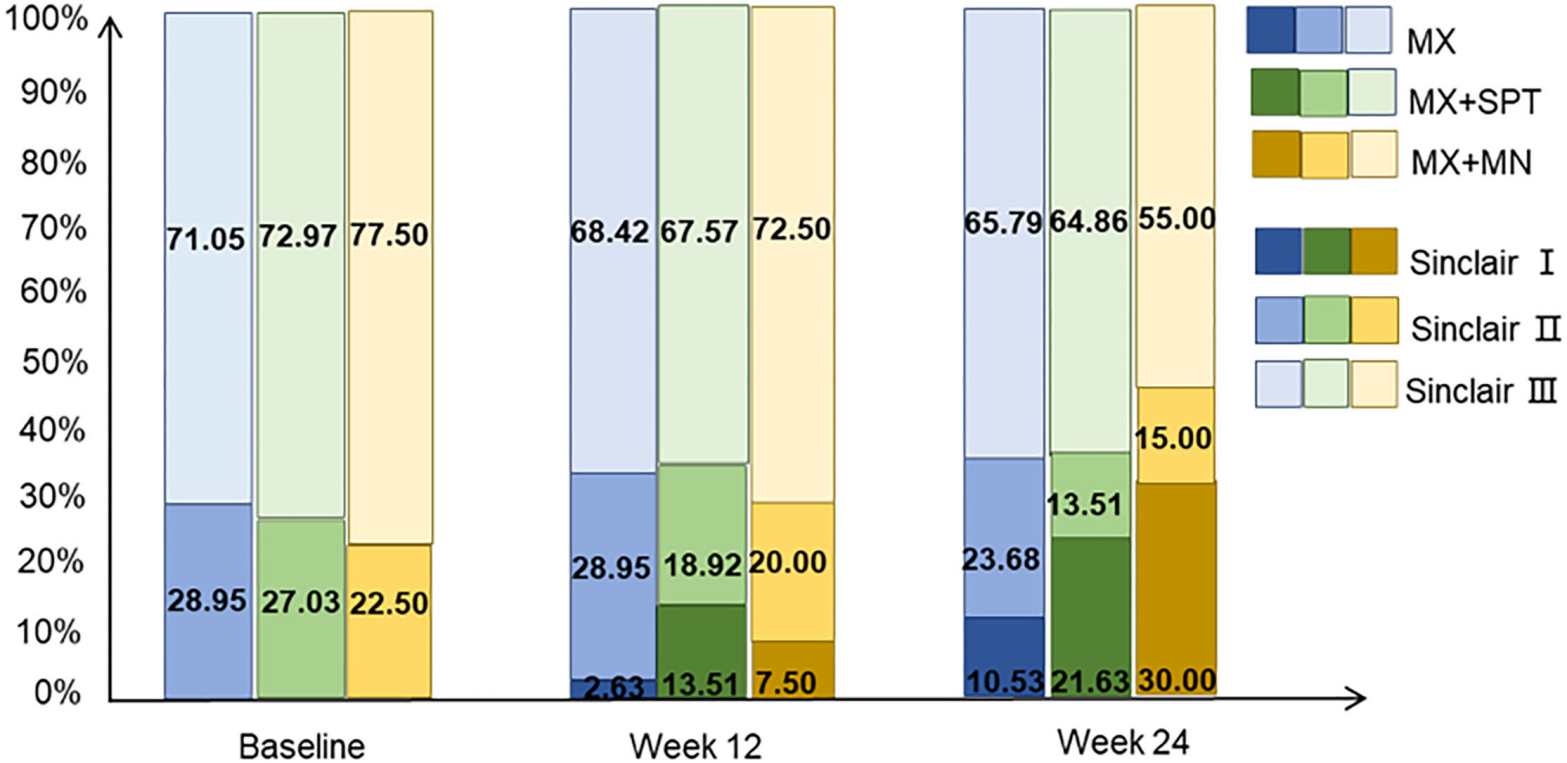
The distribution of Sinclair stage of the patients treated with minoxidil, minoxidil plus spironolactone, and minoxidil plus microneedling.

The Sinclair stage of patients among the three groups was improved during the follow-up. The change in MX + MN group was most significant. At week 24, the highest rate of Sinclair I and the minimum rate of Sinclair III were observed in this group. On the contrary, the MX group had the minimum rate of Sinclair I and the highest rate of Sinclair III. Details are shown in Figure 6.

### Patient Assessment on Hair Growth

At week 12, the hair shedding scores decreased in MX + SPT group and MX + MN group (*P* < 0.001), but not in MX group (*P* = 0.114). At week 24, the hair shedding scores decreased in all groups (*P* < 0.001). The WAA-QoL scores decreased in all groups at week 12 (*P* = 0.001, < 0.001 and < 0.001, respectively). At week 24, the WAA-QoL scores also decreased in all groups (*P* < 0.001). Details are shown in Table 4.

**Table 4:** Patients' assessment during the study period among the three groups.

**Time point**	**Median (IQR)**		
	**MX**	**MX + SPT**	**MX + MN**
**Hair shedding score**			
Baseline	4 (3–4)	4 (3–5)	4 (3–5)
Week 12	4 (3–4)	4 (2–4)	4 (2–4)
Week 24	3 (2–4)	3 (2–4)	2 (2–3)
^|^change	0 (0–1)	1 (0–1)	1 (0–1)
^§^change	1 (0–1)	1 (1–2)	2 (1–2)
^|^*P*-value	0.114	**<0.001**	**<0.001**
^§^*P*-value	**<0.001**	**<0.001**	**<0.001**
**WAA-QoL**			
Baseline	41 (28–57)	39 (27–58)	37 (28–60)
Week 12	49 (32–63)	60 (43–75)	53 (45–81)
Week 24	59 (47–77)	72 (53–89)	76 (65–92)
^|^Change	4 (1–11)	14 (5–22)	13 (7–27)
^§^Change	18 (10–25)	24 (16–39)	36 (20–43)
^|^*P*-value	**0.001**	**<0.001**	**<0.001**
^§^*P*-value	**<0.001**	**<0.001**	**<0.001**

*MX, minoxidil; SPT: spironolactone; MN, microneedling; IQR, interquartile range; WAA-QoL, women's androgenetic alopecia quality of life questionnaire.*

| *, week 12 compared with baseline;*

§ *, week 24 compared with baseline. Bold value indicates statistical significance*.

### Adverse Effects

The adverse effects were recorded in all 115 patients. The main adverse effects are listed in Table 5. Scalp pruritus was most common among the three groups and ranked first in MX + MN group (*n* = 9). The most adverse effects were reported in MX + SPT group (*n* = 45). Several adverse effects, including menstrual disorder, hyperkalemia, and edema of the limbs, only occurred in this group. The menstrual disorder was the most common adverse effect of the MX + SPT group (*n* = 15). Infection (*n* = 1) only occurred in MX + MN group.

**Table 5:** Main adverse effects during the study period among the three groups.

	**MX** **(*n* = 38)**	**MX + SPT** **(*n* = 37)**	**MX + MN** **(*n* = 40)**
Total	27	45	24
Facial hypertrichosis	4	5	5
Trichomadesis aggravating	4	4	3
Scalp pruritus	8	8	9
Increased scurf	7	6	5
Infection	0	0	1
Edema of the limbs	0	1	0
Headache	1	1	0
Palpitation	1	3	0
Postural hypotension	1	0	1
Hyperkalemia	0	1	0
Menstrual disorder	0	15	0
Urticaria	1	1	0

*MX, minoxidil; SPT, spironolactone; MN, microneedling*.

## Discussion

Topical minoxidil is the only FDA-approved drug for the treatment of FPHL. MX is a regulator of potassium ion channels with vasodilatory effects that increase the duration of the anagen phase and induce angiogenesis surrounding hair follicles, thereby contributing to the conversion of miniaturized hairs to terminal hairs (14). However, the effectiveness of MX, in general, is low in FPHL. This trial was designed to learn whether the combination therapy based on topical MX is superior to topical MX alone.

The results from our study show that the combined therapies had advantages in efficacy over topical 5% MX alone. Moreover, topical MX and MN therapy were better than topical MX and oral SPT therapy.

MX group showed improvement at week 24 in hair density and hair shaft diameter. This is consistent with a previous systematic review and meta-analysis of randomized trials, which proved the efficacy and safety of topical MX in the treatment of FPHL (15). Previous studies showed that only 46–67.7% of FPHL patients treated with topical MX could get improvement (6, 16). Likewise, our study showed that the effective rate of the topical MX alone group was only 55.27% at week 24, lower than 86.49% in MX + SPT group and 95.00% in MX + MN group. Furthermore, the degree of improvement of hair density at week 24 in the MX group was less than that in the latter two groups. Additionally, during the 1 months of topical MX treatment, some patients may suffer from transitory increased telogen hair shedding (17). Therefore, better therapy is urgently needed.

Topical MX and oral SPT of 80–100 mg daily provided improvement in FPHL patients. SPT has been proved useful in treating FPHL (18). For example, one open-label study showed that 88% of women with FPHL presented improvement or no progression after receiving mono-SPT (200 mg daily) for a minimum of 12 months (19). Burns et al. retrospectively analyzed 79 FPHL patients treated with SPT alone or in combination with topical MX or low-level laser therapy for at least 6 months, and all patients improved or maintained their initial Sinclair score (8). Apart from that, patients with an initial Sinclair score of 2.5 or higher demonstrated better improvement than those with Sinclair 1.5 or 2.0, and approximately two-thirds of patients had best recorded Sinclair score at 1 year or longer. Up to now, there are few case reports focusing on the advantage of the combination of SPT and topical MX (20). Our study showed that MX + SPT had an advantage over topical MX alone at week 24. However, the efficacy in MX + SPT group is worse than that in MX + MN group. This result may be associated with baseline hair loss severity and a short period of 24-weeks spironolactone use (8). All of our patients were Sinclair II-III at baseline and followed up for only 24 weeks, which may be the reason why they did not achieve similar or better improvement than patients in the MX + MN group.

In our study, the MX + MN group got significant improvement in most endpoints including hair density, hair shaft diameter, epidermal thickness, dermis thickness, follicle diameter, hair shedding score, and WAA-QoL score at week 24. Of note, epidermal thickness and follicle diameter were only improved in this group. The reason may be the trauma generated by needle penetration. It induces the release of platelet-derived growth factor, epidermal growth factor, and activation of the hair bulge (21, 22). Further, MN therapy activates the hair follicle stem cell proliferation and migration downward to the hair matrix through the Wnt/β-catenin pathway and contributes to the proliferation of regeneration of the scalp (23, 24). A randomized controlled study of 100 male patients demonstrated that the MN along with MX was statistically superior to MX alone in promoting hair growth in male AGA (22). Dhurat et al. also found that MN was effective for men resistant to finasteride and MX (25). In addition, the combined treatment of MN + MX was proved to be better than either treatment alone in Chinese male AGA patients (11). However, there was less evidence in the female AGA population to our knowledge. MN and topical 5% MX showed efficacy in case series of 11 treatment-recalcitrant and treatment-naive FPHL patients (9). We found that MX + MN achieved better improvement than MX alone and MX + SPT in hair density, epidermal thickness, and follicle diameter at week 24. The combined therapy of MX + MN may have two advantages over MX alone therapy. One is that MN itself is able to promote hair growth. The other is that micro-punctures caused by MN facilitate penetration of topical medications like MX (10).

Adverse effects were also necessary to be considered when choosing combination therapy. Scalp pruritus was most common in this study (21.7%, 25/115), and the frequencies in the three groups were similar. Previous studies also showed that pruritus was the most frequent dermatologic event related to 5% MX, affecting 4% of male AGA patients (26) and 5% of FPHL patients (27). Scalp pruritus resulted from multifactors, and thus 5% MX may not account for the higher frequency of pruritus observed in our patients. SPT + MX caused most adverse effects including menstrual disorder, hyperkalemia, and edema of the limbs, which only occurred in this group and had an association with SPT (28). Among the adverse effects caused by SPT, menstrual disorder ranked first. Of note, SPT is pregnancy category D, thus we should be cautious when applying SPT to women of childbearing age. Only one patient suffered from infection who was in MX + MN group. The tattoo area of this patient appeared with swelling and pain after the first MN therapy and got better soon after topical mupirocin treatment. Then, this patient completed all the following treatments and no infections occurred again. We believed that infection had an association with invasive procedures, such as MN therapy or tattoo. Hence, curable infections should not be the indication of quitting MN therapy.

In this study, Sinclair grade II and III FPHL patients with regular menstrual periods and normal hormone levels in blood were enrolled, which can avoid the effects of hormone changes on alopecia in perimenopausal and menopausal women. This study showed that MN + MX is the optimal choice for grade II and III FPHL patients with a regular menstrual period. It can avoid the side effects of oral drugs and is worth promoting in clinical application. Notably, 5% MX solution or foam once daily is recommended by FPHL guidelines, and 5% MX foam once daily has been proven to be non–inferior and as effective as 2% MX solution two times per day. The treatment of 5% MX tincture once daily for FPHL has not been reported, and we used it as one compared group in our study which may be inappropriate. Moreover, the main limitations of this study are the relatively small sample size, the exclusion of severe FPHL patients (Sinclair class for IV and V), and the inappropriate treatment of one compared group. The potential bias from unblinded treatment among the 3 groups is also a limitation in this trial. Further studies are needed.

In conclusion, considering the efficacy and safety, for mild to moderate FPHL with regular menstruation, MX + SPT is not a good choice, and MX + MN therapy is a preferable choice.

## Data Availability Statement

The raw data supporting the conclusions of this article will be made available by the authors, without undue reservation.

## Ethics Statement

The studies involving human participants were reviewed and approved by the Ethics Committee of Beijing Friendship Hospital, Capital Medical University. The patients/participants provided their written informed consent to participate in this study.

## Author Contributions

XL took charge of the statistical analysis, tables, and figures. YC wrote the manuscript. FZ revised the manuscript. All authors took part in the design and completion of the research, read, and approved the final version. All authors contributed to the article and approved the submitted version.

## Funding

This study was supported by Beijing Natural Science Foundation (No. 7222040).

## Conflict of Interest

The authors declare that the research was conducted in the absence of any commercial or financial relationships that could be construed as a potential conflict of interest. The reviewer A-hW declared a shared parent affiliation with the authors to the handling editor at the time of review.

## Publisher's Note

All claims expressed in this article are solely those of the authors and do not necessarily represent those of their affiliated organizations, or those of the publisher, the editors and the reviewers. Any product that may be evaluated in this article, or claim that may be made by its manufacturer, is not guaranteed or endorsed by the publisher.
